# Natural exosomes-like nanoparticles in mung bean sprouts possesses anti-diabetic effects via activation of PI3K/Akt/GLUT4/GSK-3β signaling pathway

**DOI:** 10.1186/s12951-023-02120-w

**Published:** 2023-09-28

**Authors:** Chengxun He, Ke Wang, Jun Xia, Die Qian, Juan Guo, Lian Zhong, Dandan Tang, Xiuping Chen, Wei Peng, Yunhui Chen, Yong Tang

**Affiliations:** 1 State Key Laboratory of Southwestern Chinese Medicine Resources, School of Pharmacy, School of Basic Medical Sciences, School of Health and Rehabilitation, CDUTCM-KEELE Joint Health and Medical Sciences Institute, Chengdu University of Traditional Chinese Medicine, Chengdu, 611137 China; 2 Hospital of Chengdu University of Traditional Chinese Medicine, Chengdu, 610072 China; 3 State Key Laboratory of Quality Research in Chinese Medicine, Institute of Chinese Medical Sciences, University of Macau, Macao, China

**Keywords:** Type 2 diabetes Mellitus, Mung bean sprouts, Exosome-like nanoparticles, Oxidative stress

## Abstract

**Background:**

Type 2 diabetes mellitus (T2DM) is a chronic metabolic disease characterized by hyperglycemia and insulin resistance. Mung bean sprouts are traditionally considered a “folk” hypoglycemic food and their pharmacological effects and underlying mechanisms warrant further investigation.

**Purpose:**

This study aimed to investigate the anti-diabetic effects of the exosomes-like nanoparticles in mung bean sprouts (MELNs) and explore the related molecular mechanisms.

**Results:**

MELNs were isolated using a differential centrifugation-polyethylene glycol (PEG) method, and the identification of MELNs were confirmed by PAGE gel electrophoresis, agarose gel electrophoresis, thin-layer chromatography (TLC), and transmission electron microscopy (TEM). In the high-fat diet/streptozotocin (HFD/STZ) mouse model, MELNs ameliorated the progression of T2DM by increasing oral glucose tolerance test (OGTT) and insulin tolerance test (ITT) results, decreasing the fasting blood glucose level, and reducing the serum triglycerides (TG) and total cholesterol (TC). Histopathological examinations indicated MELNs diminished inflammatory infiltration of hepatocytes and amplified the area of islet B cells. In addition, MELNs decreased the oxidative stress levels in liver tissue and had good biocompatibility. In vitro experiments verified that MELNs improved the viability of glucosamine (GlcN) induced insulin-resistant hepatocytes. Furthermore, this study also revealed that MELNs upregulated GLUT4 & Nrf2 and down-regulated GSK-3β via activating the PI3K/Akt signaling pathway, promoting the production of antioxidant enzymes, such as HO-1 and SOD, to reduce oxidative stress.

**Conclusion:**

MELNs mitigated the progression of type 2 diabetes in HFD/STZ mouse model. The underlying molecular mechanism is related to PI3K/Akt/GLUT4/GSK-3β signaling pathway.

## Introduction

The global prevalence of type 2 diabetes mellitus (T2DM) is alarmingly on the rise, especially within developing nations, now considered the ‘epicenters’ of T2DM. This surge is attributable to the shifts in dietary structure and lifestyle habits. In a 2010 epidemiological projection, the number of patients was expected to rise to 439 million by 2030 [1]. However, the most recent epidemiological projections in the pre-maternal period suggest a more severe situation, with type 2 diabetes accounting for nearly 90% of the 537 million cases of diabetes worldwide [2]. This number significantly surpasses the initial 2010 prediction and continues to rise. Most current treatment approaches for T2DM employ pharmaceutical agents, such as PPAR agonists, glucagon receptor antagonists, glucokinase activators, and GLP-1 agonists, among others. However, their side effects often outweigh their clinical benefits, which hampers their further development [3–6].

Exosomes are luminal vesicles formed by endosomes containing proteins, RNA, and lipids, which are eventually released into the extracellular space [7–9]. Thus, the membrane structure of exosomes and biofilms is essentially the same, comprised of a phospholipid bilayer, enhancing exosome biocompatibility. Recent studies have shown that exosomes-like nanoparticles derived from strawberries and lemons can significantly mitigate oxidative stress in human cells [10, 11]. Moreover, nanoparticles extracted from garlic have been shown to curb high-fat diet-induced inflammation in mouse brain tissue, improve glucose tolerance, and enhance insulin sensitivity [12]. Given these unique characteristics, exosomes hold tremendous potential in modulating body functions. As an affordable and readily consumable food source, plant sprouts accumulate more nutrients during germination, thus offering significant health benefits [13].

Mung bean sprouts, derived from the leguminous plant mung bean, are abundant in bioactive compounds, such as flavonoids, phenolic acids, and bioavailable amino acids [14]. Nakamura et al. found that mung bean sprout powder significantly reduces hyperglycemia induced by fructose in spontaneously hypertensive rats, along with serum triglyceride and cholesterol levels [15]. In our preliminary work, we identified exosome-like nanoparticles from mung bean sprout juice (MELNs) as potent hypoglycemic agents among various sprout extracts. In this study, we further isolated and purified MELNs, and our findings indicated that in a mouse model of type 2 diabetes, MELNs could cross cellular membranes to deliver therapeutic agents, thereby enhancing glucose transport in hepatocytes, reducing blood glucose levels, and alleviating oxidative stress caused by insulin resistance in hepatocytes. These insights may pave the way for utilizing edible plants as effective treatments for various diseases.

## Materials and methods

### Materials

The materials used in this study included fresh mung beans (acquired from a local Sam’s Club market), PEG8000 (Chron Chemicals), Nrf2, HO-1, P-Akt, Akt, GSK-3β, P-GSK-3β, GLUT4, Lamin B, β-actin (Abclone), Total cell RNA extraction kit (FORE GENE), GN5K DNA Maker (Servicebio, G3361), BCA Protein Concentration Assay Kit (BOSTER,18B06A46), SDS-PAGE protein loading buffer (BOSTER,18C20B12), Color Rapid Gel Preparation Kit 10% (BOSTER,18D11B47), reverse transcription pre-mix kit (Accurate biology, AG11728), Three-color prestained protein standards (Accurate biology, AG11919), DID (cell membrane red fluorescent dye, UElandy, 230510E01-02), BSA (BioFroxx), AST assay kit (mindray, 140,220,009), ALT assay kit (mindray, 140,120,012), SOD assay kit (Nanjing Jiancheng, A001-3-2), Glutathione peroxidase assay kit (Nanjing Jiancheng, A005-1-2), MDA assay kit (Nanjing Jiancheng), Immobilon-p PVDF(Millipore, P2231170), 4% paraformaldehyde universal tissue fixative (Biosharp, China, 23,011,108), enhanced RIPA lysate (BOSTER,18A12B02), DMED/F12(1:1) medium (Gibco, 8,123,177), DMEM medium (Gibco, 8,122,631), and a high-fat diet (containing 60 kcal% fat, Research Diets, D09100310).

### Cultivation and collection of sprouts

Fresh, well-shaped mung beans were initially selected and soaked in 37℃ water for 12 h. Well-absorbed mung beans were placed in a breathable basket, covered with wet gauze, and stored in a dark place. To maintain moisture, water was added thrice daily. Upon reaching 5 cm in length after seven days, the sprouts were collected and promptly processed to prepare MELNs.

### Isolation and purification of MELNs

The extraction method for MELNs by Zhao et al. [16] was adapted with slight modification. After washing fresh green beans with RO water, the supernatant was obtained by differential centrifugation, then the supernatant was filtered through a 1 μm filter membrane, PEG8000 (final concentration of 8%) was added, incubated at 4℃ overnight, and the precipitate was centrifuged, resuspended in an appropriate volume of PBS, sterilized through a 0.22 μm filter, and stored at -20℃.

### Characterization of MELNs

A set volume of exosome solution was dispersed in pure water, and the particle size and potential were measured using a Litesizer 500 nm particle size potential meter. Each sample underwent three parallel tests. The morphology of MELNs was observed under a transmission electron microscope (JEOL JEM-1400Plus, Tokyo, Japan) after staining freshly prepared MELNs with uranyl acetate. To further characterize the protein, RNA, and lipid existent of the exosomes, we utilized polyacrylamide gel electrophoresis, agarose gel electrophoresis, and thin-layer chromatography (TLC). After electrophoresis, the gel was stained with Coomassie brilliant blue. Lipids from MELNs were purified using the Folch method [17], separated on a silica gel TLC plate, and stained with a solution of 10% CuSO_4_ in 8% phosphoric acid.

### Cell culture

AML12 and HepG2 cell lines, procured from ATCC, were separately cultured in DMEM/F12 and DMEM (high glucose) mediums supplemented with 10% fetal bovine serum, 100 units/mL penicillin, and 100 units/mL streptomycin. The cells were incubated at 37℃ in a humidified 5% CO_2_ environment.

### Biocompatibility and cytotoxicity of MELNs

HepG2 and AML12 cells were inoculated into 96-well plates, and MELNs were diluted to a range of 25-1600 μg/mL. Cell viability was subsequently assessed at 24 and 48 h, with absorbance measured at 450 nm. In parallel, Insulin-resistant hepatocytes were created using GlcN and then exposed to the same concentration of MELNs. Cell viability and LDH release were measured. To determine the compatibility of extracellular vesicles with blood, 1ml of fresh mouse blood was mixed with extracellular vesicles at various concentrations, from 25 to 1600 μg/mL, and absorbance was recorded at 550 nm.

### Uptake of MELNs by HepG2 cells

To confirm the biocompatibility of MELNs with liver cells, we fluorescently labeled MELNs using fluorescein isothiocyanate (FITC). HepG2 cells were cultured in confocal dishes and co-incubated with 50 μl FITC-MELNs. Furthermore, HepG2 cells were inoculated into a 6-well plate and treated with FITC-MELNs for varying durations (2, 4, 8, 10 h). The cells were finally harvested for flow cytometry to measure FITC fluorescence intensity.

### Effect of MELNs on glucose uptake capacity of hepatocytes

The insulin resistance model of HepG2 cells was established by using GlcN. The cells were then treated with low (50 μg/mL), medium (100 μg/mL), and high (200 μg/mL) doses of MELNs. Subsequently, 0.3 mM 2-NBDG was added to the culture medium and incubated for 20 min. Fluorescence intensity was visualized under a confocal microscope. A glycogen assay kit was used to measure the intracellular glycogen content in the two cell lines, assessing the impact of varying MELNs doses on the glucose uptake capacity of insulin-resistant hepatocytes.

### ROS content determination in AML12 and HepG2 cells

The intracellular ROS content in HepG2 and AML12 cells modeled for insulin resistance was evaluated using the fluorescent probe CM-H_2_DCFDA. Briefly, the cells were treated with MELNs (50 μg/mL, 100 μg/mL, 200 μg/mL) for 24 h. The intracellular ROS content was detected by flow cytometry and confocal microscopy.

### Animal experiment design

Eight-week-old male C57BL/6 mice were procured from the Dashuo Experimental Animal Center (Chengdu, China). They were group-housed in miniature isolation cages under conditions of a 12-hour dark cycle, 22 °C temperature, and 50% humidity, with ad libitum access to food and water. Subsequently, mice were divided into three groups: blank, control, and model. The blank group was maintained on the NCD, while the other two groups were fed a high-fat diet for four weeks. The control and model groups were then intraperitoneally injected with streptozotocin (STZ, 100 mg/kg) [18]. The blank and model groups were administered PBS via oral gavage, whereas the control group received 40 mg/kg (dissolved in PBS) by the same method daily until the end of the fifth week. Starting from the first week, fasting blood glucose and body weight were measured weekly. In the final week, glucose tolerance and insulin tolerance were measured to evaluate the improvement of insulin sensitivity by MELNs. All animal procedures performed in this study were approved by the Animal Ethics Committee of Chengdu University of Traditional Chinese Medicine and followed their guidelines.

### Assessment of GSH-px, SOD, and MDA levels

Liver tissue was incubated with an enhanced RAPI lysis buffer at 4℃ for 30 min, then homogenized using a tissue homogenizer at 1500 rpm and centrifuged for 10 min. The levels of SOD, GSH-px, and MDA in the supernatant were determined following the instructions provided by the commercial assay kits, and absorbance was measured using a microplate reader. The contents of GSH-px, SOD, and MDA in the cells were determined similarly to the tissue. The cells were lysed with RAPI lysis buffer, and the supernatant was centrifuged as the assay solution.

### Histological analysis

Liver and pancreas tissues were fixed in 4% paraformaldehyde, embedded in paraffin, and sectioned to 5 μm thickness. After deparaffinization, the sections were stained with hematoxylin and eosin (H&E) to observe the histopathological characteristics. Similarly, deparaffinized sections were stained using the glycogen detection kit instructions, and stained tissue was observed under a confocal microscope.

### Immunofluorescence

Liver sections, post-paraffin-embedding, were deparaffinized, and the cell membrane was permeabilized with 0.5% Triton X-100. Sections were incubated with primary antibodies overnight at 4 °C, washed three times, and then incubated with secondary antibodies for one hour before imaging under a confocal microscope. Concurrently, AML12 cells were cultured in confocal dishes, removed from the incubator, and treated with DIL dye for cell membrane localization. After washing thrice, cells were fixed with 4% paraformaldehyde. Unlike the tissue protocol, there was no need to permeabilize cells with 0.5% Triton X-100 before blocking; all subsequent steps remained the same as the tissue protocol.

### Western blotting

Liver tissue was homogenized, and the supernatant was collected post-centrifugation. Protein concentration was measured using a BCA protein assay kit and normalized. Protein separation was achieved through SDS-PAGE and transferred onto a polyvinylidene fluoride (PVDF) membrane. Cell proteins were extracted by incubation with RAPI lysis buffer for 30 min, followed by the same procedures as the animal protein extraction. Primary antibodies including anti-β-actin (1:800), anti-Lamin B (1:1000), anti-Phospho-Akt (1:1000), anti-Akt(1:1000), anti-GLUT4 (1:1000), anti-Phospho-GSK-3β (1:1000), anti-GSK3β (1:1000), anti-HO-1 (1:1000), anti-Nrf2 (1:1000), anti-GS (1:1000), anti-Phospho-GS (1:1000), anti-Phospho-PI3K (1:1000), and anti-PI3K (1:1000) were used. After overnight incubation at 4 °C, membranes were washed thrice with TBST and incubated with secondary antibodies for one hour. Protein bands were visualized using ECL reagent and quantitatively analyzed with ImageJ software.

### Statistical analysis

All statistical analyses were carried out using SPSS version 19.0 (IBM, Chicago, USA) software. The average value of all experimental data is presented as mean ± SD, and one-way analysis of variance (ANOVA) was used to determine the significance of the results. A P-value of < 0.05 was considered statistically significant.

## Results

### Characterization of mung bean exosome-like nanoparticles (MELNs)

**Fig. 1:**
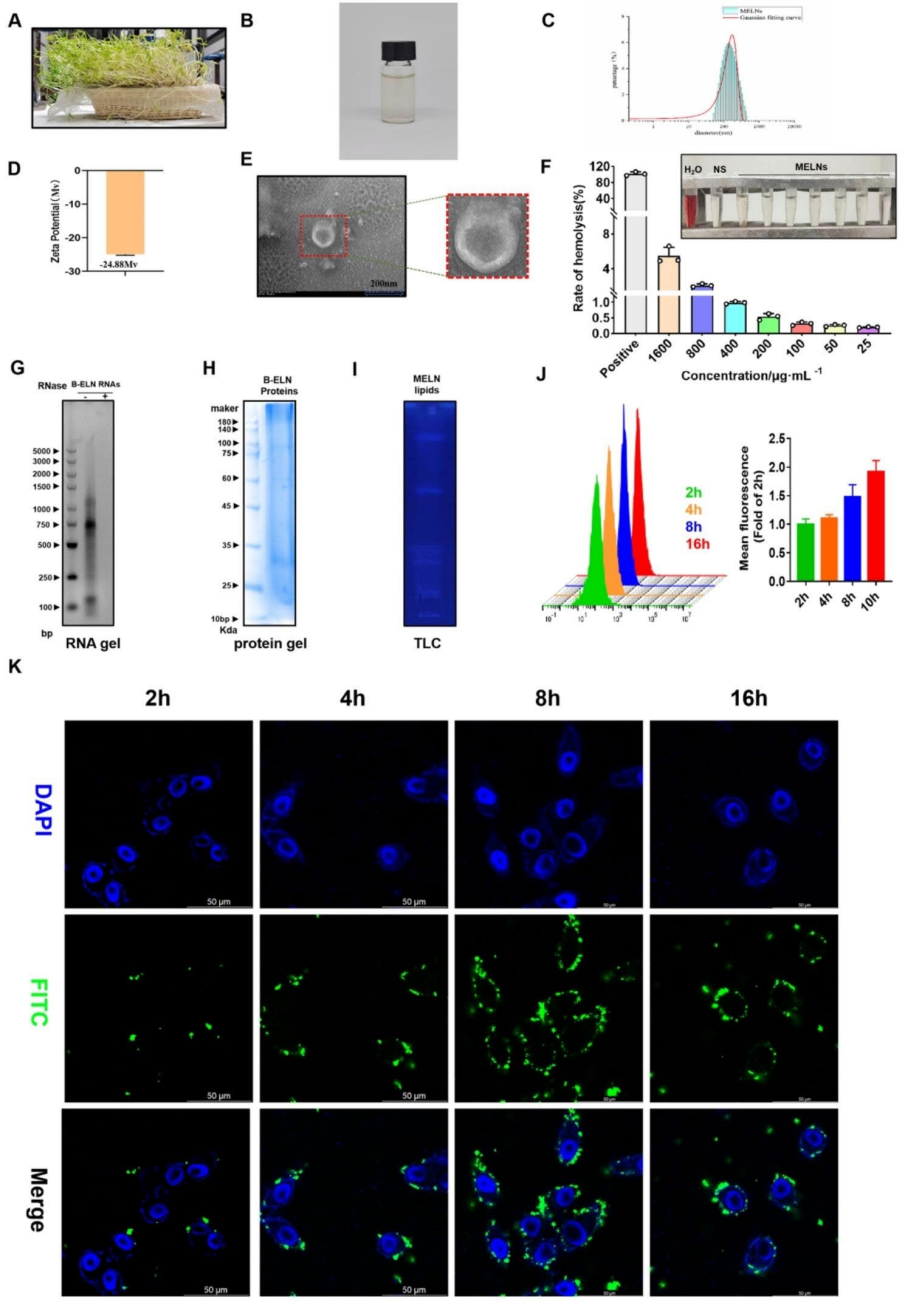
Characterization of MELNs. (**A**)-(**B**) The appearance of bean sprouts and MELNs. (**C**)-(**D**) Particle size of MELNs as well as Zeta potential. (**E**) TEM. (**F**) Effect of MELNs on blood cells. (**G**)-(**I**) Proteins, lipids, and nucleic acids in MELNs. (**J**)-(**K**) Uptake of MELNs by hepatocytes

MELNs were isolated from mung bean sprouts seven days after planting and dispersed in PBS (Fig. 1A&B). Laser particle size analysis revealed an average particle size of 149.23 nm and a zeta potential of -24.88Mv (Fig. 1C&D). The electron microscopy confirmed the particle morphology. According to the scale, the particle size was about 150 nm, which was consistent with the data measured by the laser particle size analyzer (Fig. 1E).

Subsequently, after MELNs were incubated with blood cells, the results showed in Fig. 1F that even if the concentration reached 1600 μg/ml, the hemolysis rate only reached 5.44%, and the hemolysis rate gradually decreased with the decrease of the dose. SDS-PAGE analysis stained with Coomassie Brilliant Blue showed protein sizes ranging from 10 ~ 100Kda within the MELNs (Fig. 1G). The presence of RNA enzymes confirmed that the MELNs contain RNA, primarily distributed within a molecular weight range of 100-1500 bp (Fig. 1H). Furthermore, TLC data showed that MELNs encompass multiple types of lipids (Fig. 1I). Thus, the MELNs examined in this study exhibit exosome-like properties.

### MELNs mitigate progression of STZ-induced type 2 diabetic mice

**Fig. 2:**
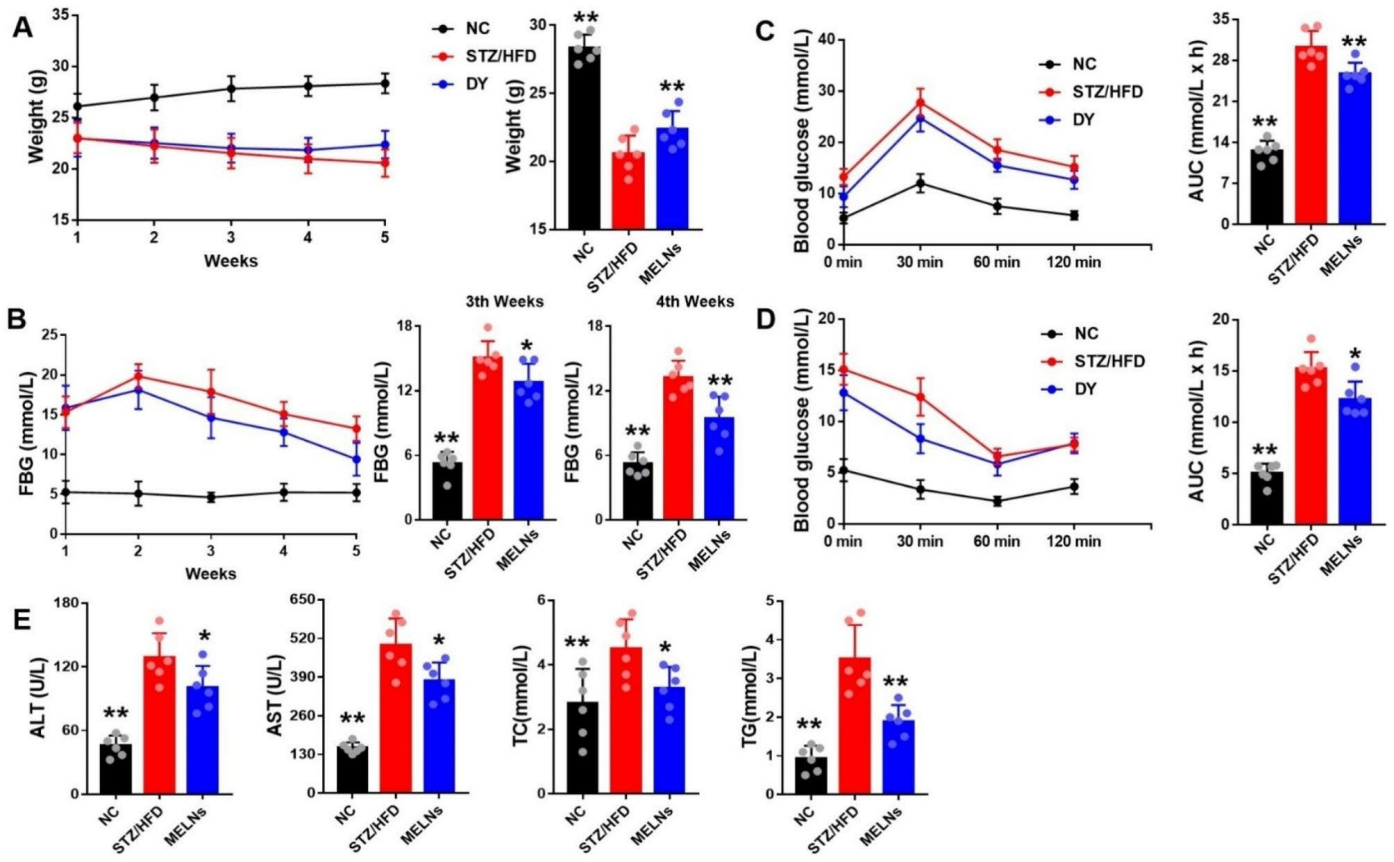
Progression of type 2 diabetes in HFD/STZ mice. (**A**) Changes in body weight in mice. (**B**) Changes in fasting blood glucose in mice at the third and fourth weeks. (**C**)-(**D**) Changes of OGTT and ITT in mice. (**E**) Serum levels of TG, TC as well as AST and ALT. **p < 0.01, *p < 0.05 compared to the HFD/STZ mice

As noted in previous studies, the STZ-treated mice showed weight loss, elevated fasting blood glucose, increased lipid levels, and diminished glucose tolerance [19, 20]. However, MELNs administration mitigated these conditions (Fig. 2A&B). Among them, the glucose and insulin tolerance tests showed that the diabetic mice presented a significantly higher area under the curve than that of the normal group, and the area was significantly reduced upon MELNs intervention (Fig. 2C&D). MELNs also lowered serum AST/ALT levels and significantly reduced TG and TC content compared to the model group (Fig. 2E).

### Effects of MELNs on liver tissue and islet B cells in diabetic mice

**Fig. 3:**
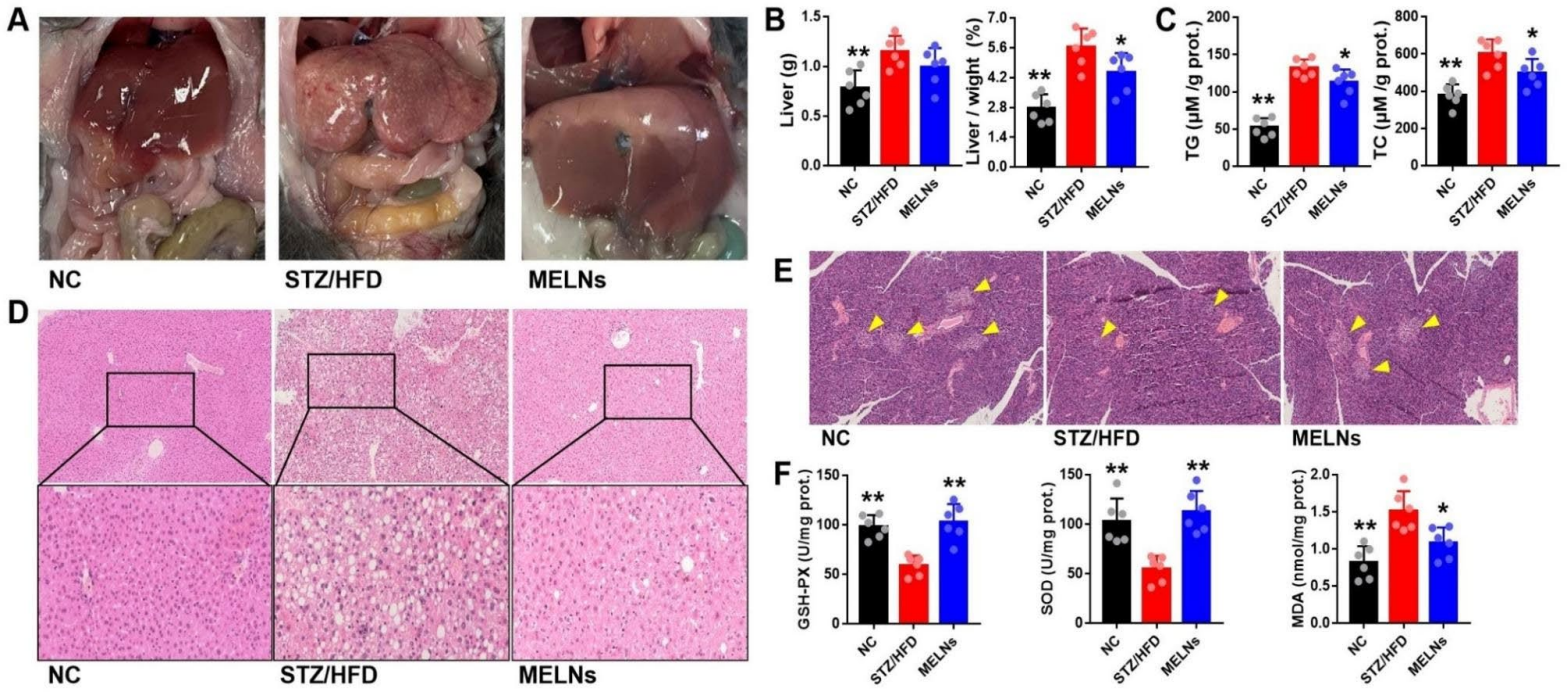
MELNs improved liver pathological changes, lipid accumulation and function. (**A**)-(**B**) Liver morphological changes and organ index, *p < 0.05, compared to the HFD/STZ mice. (**C**) TG, TC levels in liver tissue. (**D**)-(**E**) HE staining of the liver and pancreas was performed. (**F**) Function parameters of liver. **p < 0.01, *p < 0.05 compared to the HFD/STZ mice

As illustrated in Fig. 3A&B, MELNs significantly improve the liver morphology of STZ/HFD diabetic mice and reduce the liver organ index, and no apparent changes are noted in liver weight. As shown in Fig. 3C, the levels of TG and TC in liver tissue were significantly reduced with MELNs administration. The H&E staining of the liver and islets further elucidated that MELNs could reduce inflammatory invasion of liver and increase the number of islet B cells in the model group (Fig. 3D&E). In addition, as presented in Fig. 3F, the GSH-px and SOD activities in MELNs-treated mouse liver tissues significantly differed from those in the model group (P < 0.05). The MDA content in the normal group was significantly lower than that in the model group, while MELNs-treated mice showed a significant decrease in liver tissue MDA content.

These results confirm that MELNs can mitigate liver and islet damage and reduce oxidative stress levels in STZ/HFD mice. However, the precise molecular mechanism remains to be elucidated. Therefore, we utilized GlcN to create insulin-resistant hepatocytes.

### Biocompatibility and in vitro antioxidant activity of MELNs

**Fig. 4:**
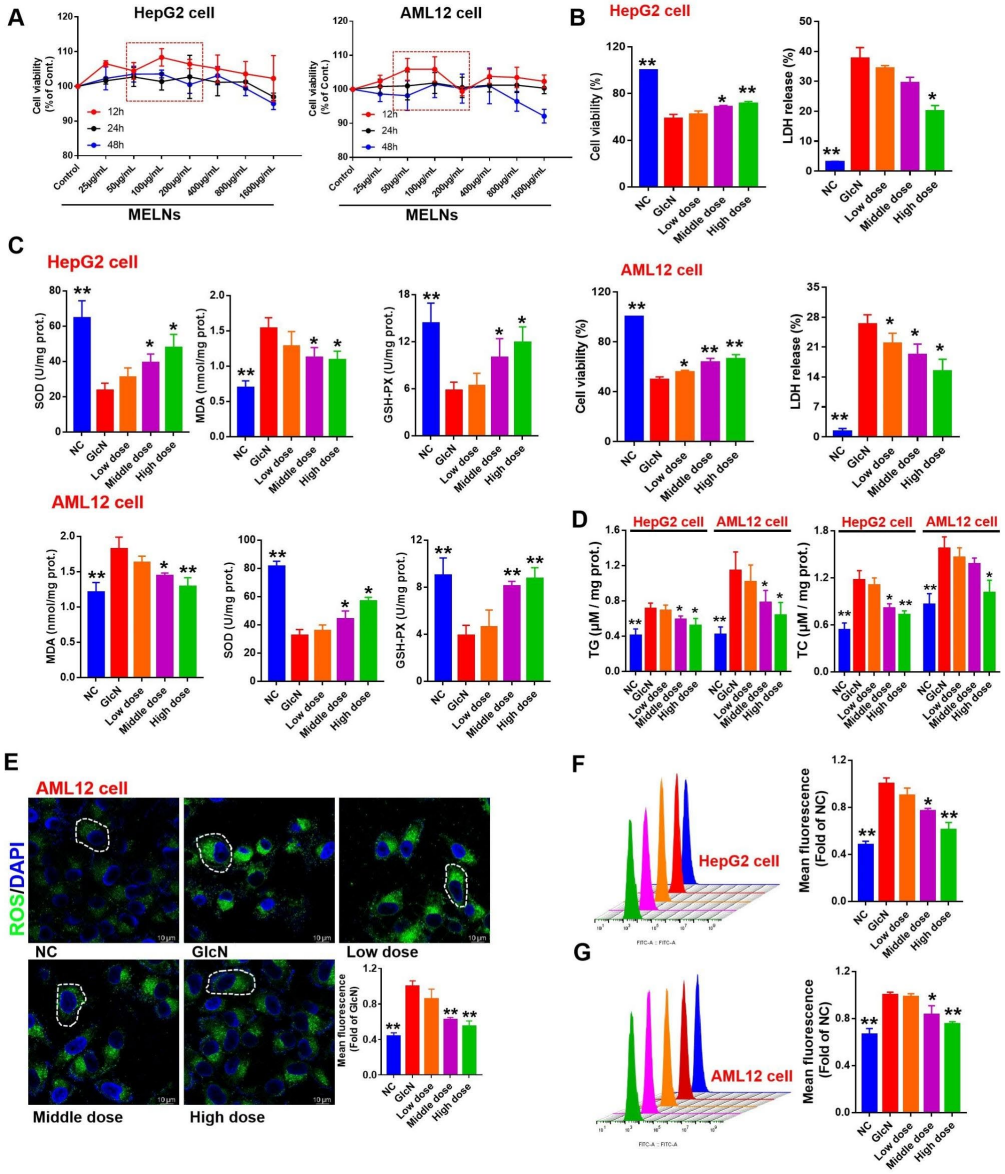
MELNs did not significantly reduce the viability of hepatocytes and reduced the oxidative stress level after Glcn-induced insulin resistance. (**A**)-(**B**) CCK8 assay was used to determine the effect of MELN on the cell viability of normal hepatocytes and GlcN (+) hepatocytes. (**C**)-(**D**) Parameters of hepatocyte function. (**E**)-(**G**) Changes of intracellular ROS in GlcN (+) hepatocytes treated with MELN. **p < 0.01, *p < 0.05 compared to the GlcN (+) hepatocytes

The CCK-8 assay results, depicted in Fig. 4A, revealed that after co-incubation with MELNs, cell viability exhibited a dose-dependent and time-dependent decrease. However, under all dose and time conditions, cell survival rates remained above 90%. To ensure the rigor of subsequent experiments, we selected 50, 100, and 200 μg/ml as the dosages. The presence of GlcN reduced the activity of liver cells (P < 0.01), and MELNs attenuated this change (P < 0.01). Similarly, the release rate of LDH decreased significantly with the increase of MELNs dose (P < 0.05) (Fig. 4B).

In addition, we observed the uptake of MELNs by HepG2 cells. As shown in Fig. 1K, the amount of FITC-MELNs in the field of view first increased and then decreased with increasing uptake time.Over time, the intensity gradually decreased due to fluorescence quenching. This idea was further confirmed by flow cytometry results, which showed that the fluorescence intensity detected gradually increased with time (Fig. 1J).

### Reduction of GlcN-Induced oxidative stress levels in liver cells by MELNs

In this study, we initially measured the levels of triglycerides (TG) and total cholesterol (TC) in two hepatocyte lines. Our results, depicted in Fig. 4D, indicate that compared to the control group, both TG and TC levels increased significantly in the model group (P < 0.01). Intriguingly, the TG and TC levels decreased after co-incubation with GlcN in both the medium and high-dose MELNs group (P < 0.05).

We further measured the levels of SOD, GSH-px, and malondialdehyde (MDA) in the cell homogenate. As illustrated in Fig. 4C, MDA levels substantially increased in insulin-resistant hepatocytes (P < 0.01). Conversely, after inducing insulin resistance in liver cells, antioxidant enzyme activities, represented by SOD and GSH-px, decreased. MELNs treatment could restore a certain amount of enzyme activity, especially in HepG2 cells, where a significant difference was noted between the medium and high doses (P < 0.05). For AML12 cells, MELNs exhibited a more pronounced effect on enzyme activity, particularly for GSH-px, with a substantial difference between the medium and high doses (P < 0.01). The difference in SOD levels between the medium and high doses, however, was not significant (P < 0.05).

Then, we determined the ROS level under the co-incubation of GlcN and MELNs. After adding MELNs, ROS (green fluorescence) in the cells was significantly decreased (P < 0.05) (Fig. 4E). Flow cytometry results confirmed that GlcN increased ROS content in both liver cell lines (Fig. 4F&G). As the MELNs dose increased, fluorescence intensity reduced, indicating a gradual decrease in ROS content. The high-dose MELNs group displayed a significant difference in comparison to the model group (P < 0.01).

**Fig. 5:**
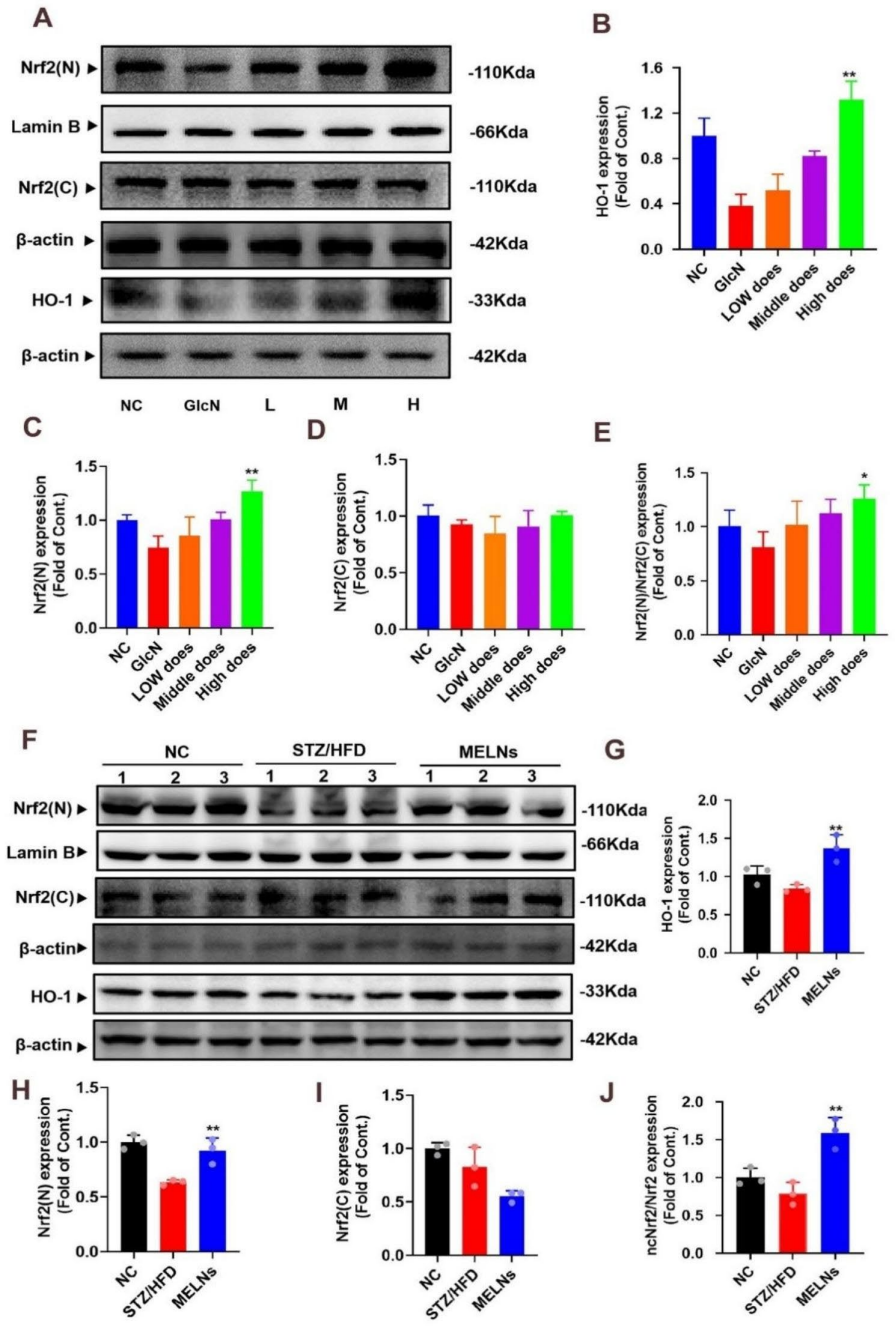
Expression of oxidative stress-related proteins. (**A**)-(**E**) Western blotting assays showed that MELNs promoted Nrf2 nuclear translocation and increased HO-1 expression. **p < 0.01, *p < 0.05 compared to the GlcN (+) hepatocytes. (**F**)-(**J**) In vivo experiments also showed that the expression level of HO-1 and the ratio of Nrf2(N)/Nrf2(C) expression were increased after administration of MELNs. **p < 0.01, *p < 0.05 compared to the HFD/STZ mice

As we demonstrated earlier, MELNs can enhance the activity of SOD and GSH-px antioxidant enzymes. However, the molecular regulatory mechanism of their upstream pathway remains to be clarified. We employed Western blots to measure the expression of relevant proteins, including HO-1 and Nrf2, in HepG2 cells. Our findings (Fig. 5A) indicated a decrease in HO-1 and nuclear Nrf2 protein expression in insulin-resistant HepG2 cells. However, MELNs administration reversed these effects in a dose-dependent manner. Notably, HO-1 expression in the high-dose group differed significantly from the model group (P < 0.05), as depicted in Fig. 5B.

In contrast, there was no significant change in cytoplasmic Nrf2 levels, aligning with previous literature [21]. But we noted a significant increase in nuclear Nrf2 (P < 0.05) (Fig. 5C). We also investigated HO-1 and Nrf2 protein expression levels in liver tissues. Interestingly, in vivo, cytoplasmic Nrf2 expression in the model group was marginally lower than in the normal group (Fig. 5I). The cytoplasmic Nrf2 expression in MELNs-treated mice continued to decrease, but the difference was not statistically significant compared to the model group(P > 0.05). The rest of the results were consistent (Fig. 5F). HO-1 and Nrf2 expression in liver tissues increased notably with an increase in MELNs dose in vivo (P < 0.01) (Fig. 5G&H).

### Nrf2 activation through PI3K/Akt/GSK-3β pathway

**Fig. 6:**
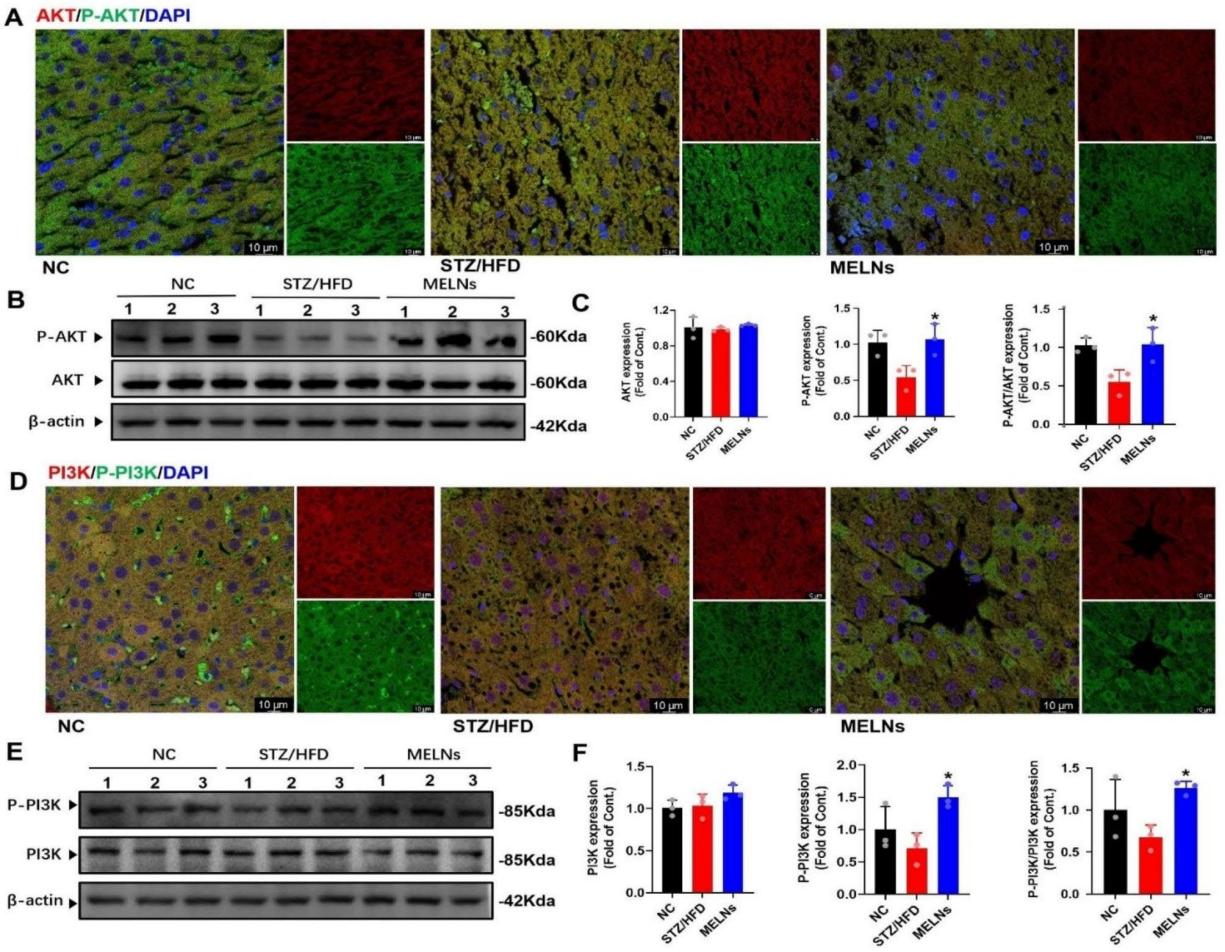
Expression of PI3K/Akt in tissues. (**A**)(**D**) The expression of P-Akt/Akt and P-PI3K/Akt was detected by immunofluorescence. (**B**)-(**C**) Western blotting results showed that MELNs increased the phosphorylation of Akt and PI3K. (**E**)-(**F**) in the tissues. *p < 0.05 compared to the HFD/STZ mice

**Fig. 7:**
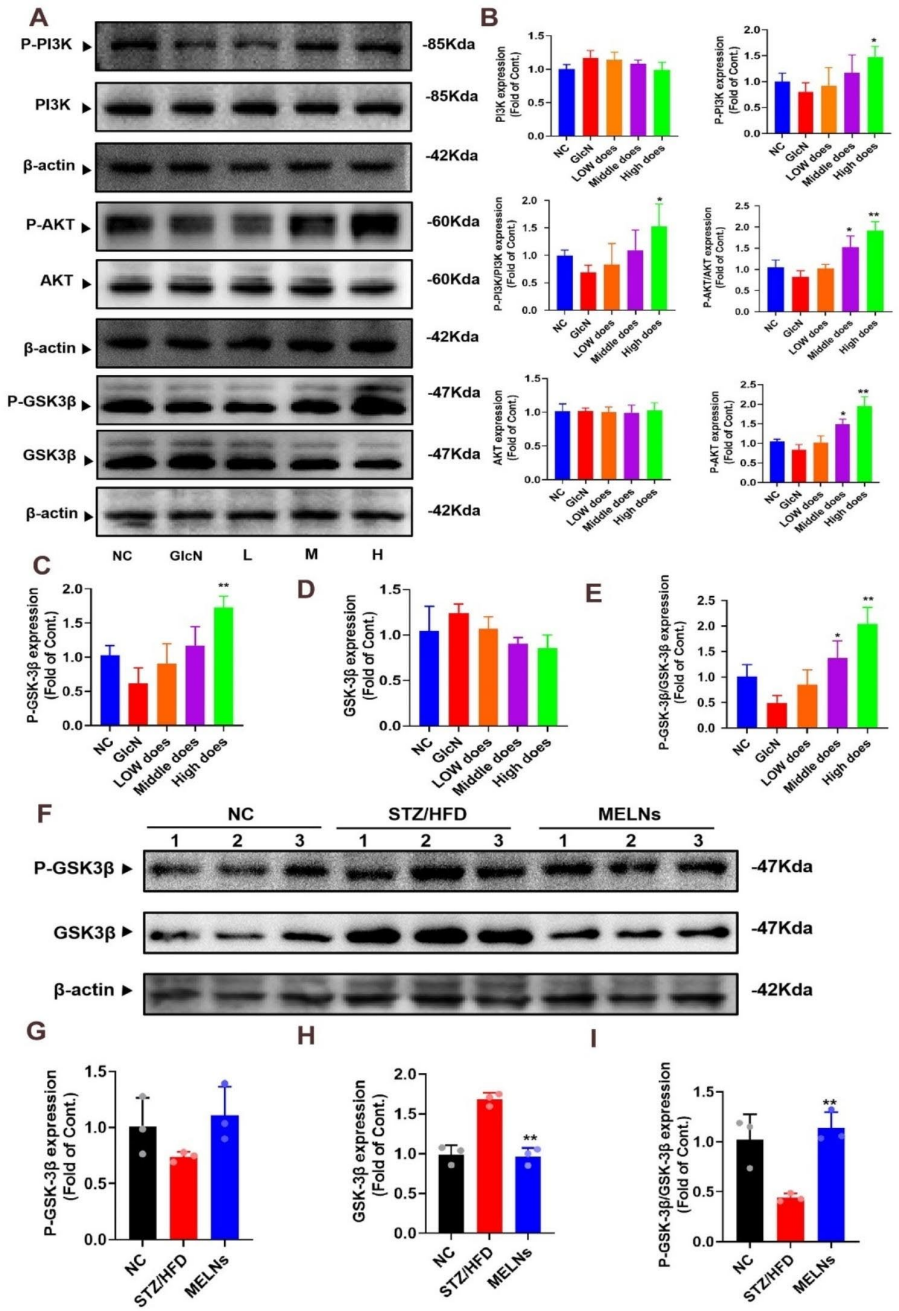
Protein expression of PI3K/Akt/GSK-3β pathway. (**A**)-(**E**) Expression of PI3K/Akt/GSK-3β and their phosphorylated proteins in hepatocytes in vitro **p < 0.01, *p < 0.05 compared to the GlcN (+) hepatocytes. (**F**)-(**I**) The protein expression of P-GSK-3β/GSK-3β in liver tissue was detected. **p < 0.01, *p < 0.05 compared to the HFD/STZ mice

Immunofluorescence results revealed that PI3K and Akt were primarily expressed in the cytoplasm, and phosphorylation levels of both proteins decreased after STZ/HFD modeling. Interestingly, phosphorylation levels of both proteins recovered to some extent after MELNs were administered (Fig. 6A&D). Western blotting (WB) results echoed these findings (Fig. 6C&F), showing that MELNs significantly amplified the phosphorylation levels of Akt and PI3K, with a statistically significant difference (P < 0.05). Similarly, the same protein expression trends were observed at the cellular level and in vivo (Fig. 7A&B).

Notably, GSK-3β expression decreased progressively with rising MELNs dosage (Fig. 7C-E), while P-GSK-3β expression increased. This indicates that MELNs promoted GSK-3β phosphorylation, thereby reducing its activity as a negative Nrf2 regulator, subsequently easing its inhibitory effect on Nrf2. In vivo experiments mirrored the same cellular level results (Fig. 7F-I). These results substantiate the assertion that the activation of Nrf2 and its associated antioxidant enzymes is mediated by the PI3K/Akt/GSK-3β signaling pathway.

### MELNs enhance glucose absorption and foster glycogen synthesis

**Fig. 8:**
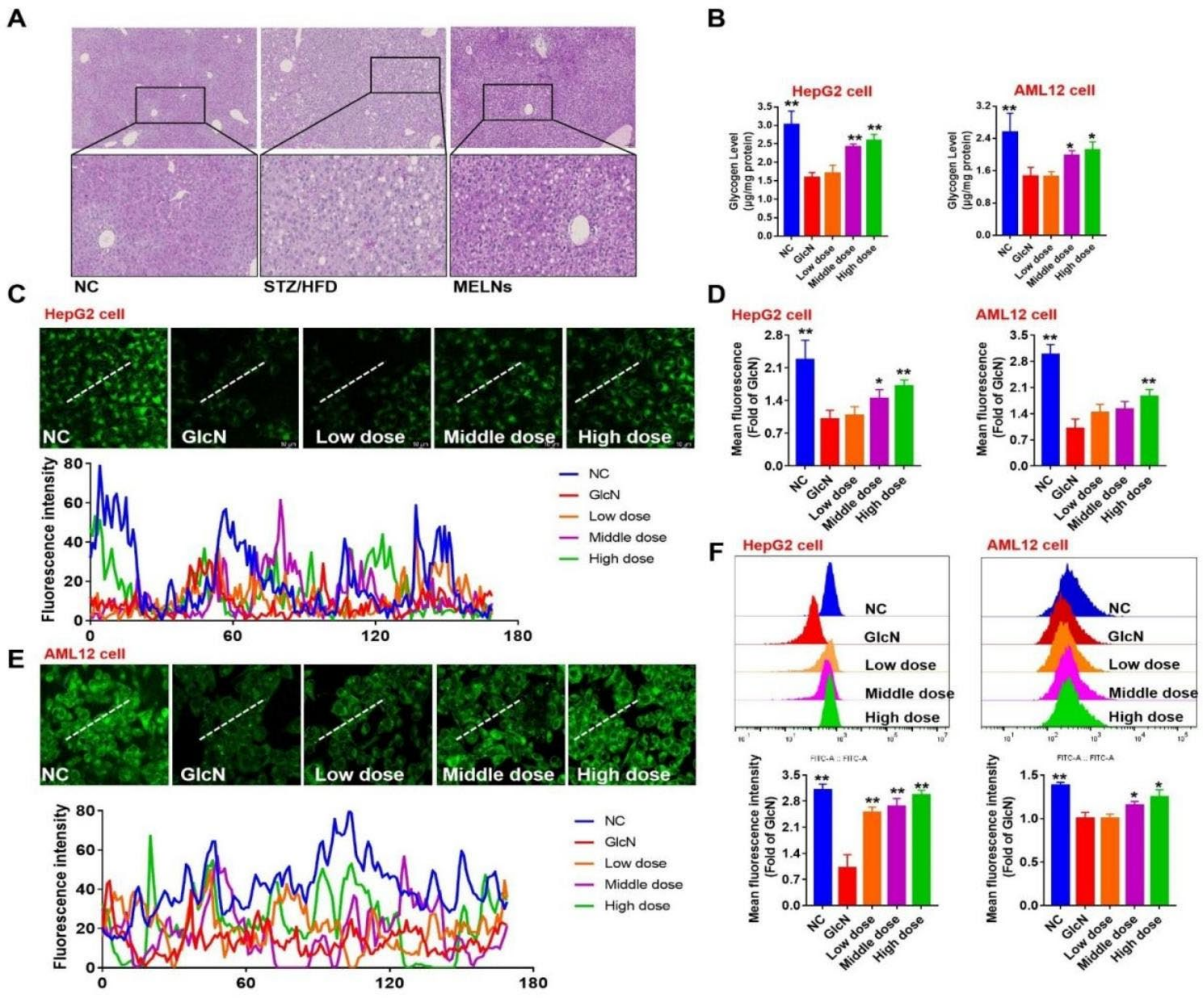
Changes in glycogen accumulation in liver tissue as well as assessment of glucose uptake capacity. (**A**) Accumulation of hepatic glycogen in tissues. (**B**) Glycogen changes in hepatocytes after GlcN induction. (**C**)-(**E**) Uptake of 2-NBDG by hepatocytes. (**F**) Flow cytometry was used to determine the ability of hepatocytes to absorb 2-NBDG. **p < 0.01, *p < 0.05 compared to the GlcN (+) hepatocytes

PAS staining was employed to assess the effect of MELNs on the accumulation of glycogen in tissues. The results showed that MELNs significantly increased the accumulation of glycogen in tissues and elevated the glycogen content in liver cells after GlcN modeling (Fig. 8A&B) (P < 0.05). We then evaluated the effect of MELNs on glucose uptake of cells, and the results showed that MELNs could increase the fluorescence intensity of GLcN compared with the model group (Fig. 8C&E). After quantification of colocalization results, the difference was significant (Fig. 8D) (P < 0.05). Similarly, flow cytometry (Fig. 8F) also demonstrated that medium and high MELNs doses significantly increased glucose uptake in a GlcN-induced insulin resistance model (P < 0.05).

**Fig. 9:**
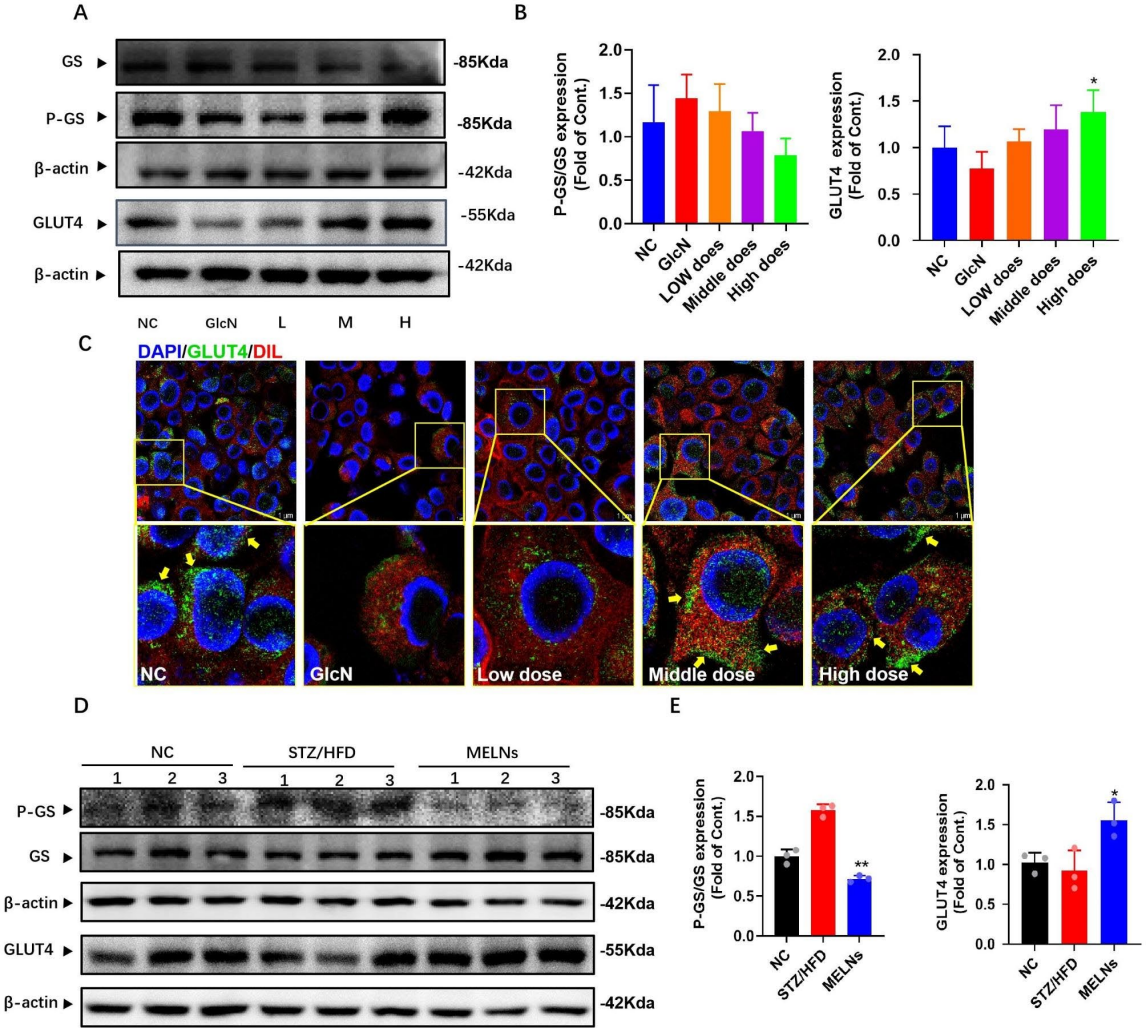
MELNs increased membrane translocation of GLUT4 and protein expression levels of GS and GLUT4. (**A**)-(**B**) MELNs increased the protein expression of GLUT4 and GS in hepatocytes in vitro. **p < 0.01, *p < 0.05 compared to the GlcN (+) hepatocytes. (**C**) Expression of GLUT4 at the cell membrane. (**D**)-(**E**) Expression of GS and GLUT4 in vivo. **p < 0.01, *p < 0.05 compared to the HFD/STZ mice

MELNs administration restored GS phosphorylation to some extent, but the difference was not statistically significant (P > 0.05). GLUT4 receptor on the cell membrane determines glucose uptake. Immunofluorescence and Western blotting revealed (Fig. 9A&B) that GLUT4 protein expression was decreased in GlcN-induced insulin-resistant AML12 cells. However, GLUT4 expression increased after MELNs administration, with a significant difference compared to the model group (P < 0.05).

The immunofluorescence was employed to label GLUT4 on the membrane and DID membrane dye for cell membrane localization. The results showed (Fig. 9C) that the green fluorescence on the membrane intensified with increasing MELNs dosage, indicating enhanced GLUT4 expression. Compared with the blank group, the results of the model group were consistent with the WB results. The expression of GLUT4 in the cell membrane of the model group was significantly decreased (P < 0.05). We also measured the expression of GLUT4 and GS protein in the tissues, and the results were shown in the Fig. 9D. Unlike in vitro experiments, MELNs significantly (Fig. 9E) enhanced the phosphorylation level of GS (P < 0.01) and also significantly enhanced the phosphorylation level of GLUT4 (P < 0.05). These results suggest that MLENs increase the expression of GLUT4, promote glucose uptake, and enhance the glycogen synthesis ability of cells.

## Discussion

The global prevalence of diabetes is surging at an alarming pace, particularly in developing nations [2]. In Asia, the epicenters of the disease are China and India, where populations exhibit a younger age of onset, a lower BMI at onset, and higher incidences of complications such as stroke and kidney disease than Caucasian populations [22]. Presently, the primary treatment for T2DM is pharmacotherapy, which includes GLP1 modulators, Thiazolidinedione insulin sensitizers, and SGLT2 inhibitors, among others [23]. While these treatments are often paired with dietary and exercise regimes to mitigate T2DM impact [24], the associated side-effects are non-negligible [5–8]. In addition, as economic globalization continues to evolve, time constraints exacerbate challenges in managing lifestyle factors effectively.

Strategies to lower blood glucose primarily focus on enhancing the glucose route and minimizing glucose absorption. Recently, an array of natural products demonstrating potential in treating type 2 diabetes have been discovered, and their mechanisms of action are currently under investigation [25, 26]. The discovery of these natural products suggests that certain plant components or constituents can exhibit pharmacological effects in the human body with promising biocompatibility, drug resistance, and safety. Recently, edible plant exosomes-like nanoparticles have been identified as potential treatments for a range of diseases. For example, ginger exosome-like nanoparticles can alleviate alcohol-induced liver damage and reduce oxidative stress through the activation of Nrf2 via the TLR4/TRIF pathway [27].

This study isolated edible MELNs from mung bean sprouts as a novel component comprising lipids, proteins, and RNA. Initially, we assessed the biocompatibility of MELNs, and the results demonstrated that MELNs did not significantly reduce cell activity at higher concentrations. MELNs could release substances into cells via the cell membrane, showing similar results to other edible ELNs, such as blueberries, strawberries, and ginger [11, 16, 28]. This trend can be attributed to cells’ uptake of extracellular vesicles, which are anchored on the cell membrane and absorbed into the cytoplasm through endocytosis [29]. These plant ELNs have also exhibited good biocompatibility. Researchers have further explored these exosomes’ composition, isolating and purifying proteins, lipids, and nucleic acids contained within them. For instance, Ju et al. [30] performed lipidomic analysis of grapefruit exosome-like nanoparticles, identifying 11 lipids, of which phosphatidic acid (PA) constituted 53.2%. Chen et al. [31] also conducted lipidomic analysis of ELNs and identified 10 lipids, with the ratio of PA reaching 40.41%. This evidence suggests that ELNs membrane structure may consist of a phospholipid bilayer, with phospholipid macromolecules possibly hydrolyzed to phosphoric acid, glycerol, and other substances by phospholipase during the extraction process.

In commonly used HFD/STZ-induced type 2 diabetes animal models, our study discovered that MELNs significantly attenuated T2DM progression. This was evidenced by restoration of body weight, enhanced glucose tolerance and insulin sensitivity, and reduced serum TG and TC levels. These alterations indicate that MELNs exert substantial therapeutic effects on STZ/HFD mice. Remarkably, MELNs led to a reduction in the basal blood glucose of HFD/STZ mice. From the appearance of the tissue, the color of the model group was earth yellow, which was caused by the accumulation of too much triglycerides in the liver. After MELNs were given, the color of the liver was changed, and the surface changed from rough to smooth, suggesting that it presented protective effects on the liver. Upon observation of the H&E staining of liver and pancreatic tissue, we found that MELNs could reduce hepatocyte inflammatory infiltration, decrease local tissue vacuolization, and increase the islet cell population area in the pancreatic tissue. This suggests that MELNs could potentially stimulate the proliferation of islet B cells. However, this study did not confirm the protective effect of MELNs on islet B cells.

Following the modeling process, the liver appeared hypertrophic, with its color transitioning from dark red to grayish-yellow, aligning with prior literature reports [32, 33]. After administering MELNs, these indices showed signs of recovery. After observing changes in the liver tissue, we measured the levels of GSH-px, SOD, and MDA in the liver tissue and found a significant increase in the activities of two antioxidant enzymes post-MELNs administration. The activation of the antioxidant enzyme system requires the nuclear translocation of Nrf2 [34], prompting us to conduct a WB analysis of the liver tissue. The findings suggested that MELNs facilitated Nrf2 nuclear translocation and significantly elevated HO-1 expression, leading us to hypothesize that MENLs could reduce oxidative stress.

To further unravel the mechanism through which MELNs mitigate disease progression in T2DM mice, we generated an insulin-resistant hepatocyte model using Glucosamine (GlcN), a substance extensively utilized to simulate insulin resistance in cellular models [35, 36]. Our findings revealed increased MDA and LDH levels, coupled with decreased SOD and GSH-px activities in the insulin-resistant hepatocyte lines. This data implies that the modeling method also elevates oxidative stress levels in hepatocytes. Notably, co-incubation with MELNs reduced oxidative stress levels, underscoring MELNs’ protective effect on insulin-resistant hepatocytes.

In line with the in vivo study, we also examined the expression of Nrf2-related proteins in cells. Nrf2, a transcription factor, regulates the secondary antioxidant system. Prior to oxidative stress, Nrf2 is anchored in the cytoplasm by Keap1, which continually suppresses its activity [37]. Oxidative stress triggers ROS or oxidizing/electrophilic molecules to modify Keap1’s cysteine residues, prompting Nrf2’s release [38]. Following its nuclear entry, Nrf2 binds to antioxidant response elements, thereby inducing expressions of heme oxygenase-1 (HO-1), superoxide dismutase-1 (SOD1), NAD(P)H quinone oxidoreductase-1 (NQO1), among other oxidases. When these antioxidant enzymes are translated, they can remove ROS, MDA, and other substances that damage cells in the cytoplasm and play a protective role.

In vitro experiments indicated that MELNs facilitated Nrf2 nuclear translocation and augmented HO-1 expression, suggesting that MELNs alleviate oxidative stress in GLN-induced insulin resistance in hepatocytes. To understand how MELNs modulate the oxidative stress system, we explored the system’s upstream pathway via experimental techniques. The Keap1/Nrf2 pathway traditionally regulates REDOX balance, but we did not evaluate Keap1 expression in our study. The reason is that from the consequence of glycogen staining and glucose uptake, the regulation of Nrf2 antioxidant oxidase system may be its non-classical pathway, that is, through GSK-3β to regulate Nrf2 entry into the nucleus. Glycogen synthase kinase 3β (GSK-3β) is deemed a common effector of numerous Nrf2 inducers in managing the Nrf2 non-canonical pathway, independent of Keap1 [39]. GSK-3β, a protein that modulates Nrf2 oxidative stress levels, plays a pivotal role in numerous diseases [40, 41]. As a negative regulator of Nrf2, GSK-3β sees a reduction in protein activity upon phosphorylation, with protein activity decreasing proportionally to the degree of phosphorylation.

In vivo, we observed that MELNs reduced blood glucose and enhanced glucose tolerance. We postulated that the blood glucose reduction might be linked to increased glucose efflux. In the liver, glycogen synthesis significantly contributes to increased glucose efflux. However, elevated oxidative stress levels in insulin-resistant hepatocytes block glycogen synthesis [42, 43]. At the protein level, MELNs were shown to increase the phosphorylation of GSK-3β, thus inactivating GSK-3β and preventing it from reversely regulating Nrf2. Interestingly, GSK-3β regulates glycogen synthesis. Liver glycogen synthesis is one of the main ways to reduce blood sugar levels. When glycogen synthesis is blocked, hyperglycemia will be aggravated [44, 45]. Typically, GSK-3β triggers GS phosphorylation, thus reducing GS activity and decreasing glycogen synthesis. Our study showed that MELNs incubation decreased GSK-3β expression, elevated P-GSK-3β, and increased GS expression, promoting glycogen synthesis. PAS staining confirmed that glycogen synthesis significantly increased in AML12 cells after MELNs incubation.

Given this evidence, we chose GSK-3β as our study’s signal transduction hub. Increased GS expression leads to a glycogen boost in hepatocytes; we then questioned whether MELNs could enhance glucose uptake. In insulin-resistant hepatocytes, the efficiency of glucose utilization and uptake is diminished [41, 46]. GlcN escalated GS phosphorylation, which generally loses its activity post-phosphorylation and is thus unable to synthesize glycogen and can affect glucose uptake [47]. Given that glucose uptake is critical for glycogen synthesis and is inhibited in insulin-resistant cells [48], we evaluated the glucose uptake capacity of hepatocytes. We found that MELNs notably augmented intracellular glucose content compared to the model group, with glucose uptake dependent on membrane GLUT4. Hence, we utilized WB and immunofluorescence to show that MELNs boosted the membrane translocation of GLUT4. For more effective proof, only total protein was extracted to incubate GLUT4 in our experiment, but future work should involve extracting cell membrane proteins to incubate GLUT4.

In hepatocytes experiencing insulin resistance, the regulation of insulin signal transduction becomes compromised, and the PI3K/Akt signaling pathway, downstream of the insulin receptor, is suppressed. This pathway acts as a fundamental node of insulin signal transduction, regulating glucose uptake, cellular metabolism, and glycogen synthesis. It plays a pivotal role in managing insulin resistance and hyperglycemia in type 2 diabetes [49]. Researchers have discovered that the PI3K/Akt pathway can activate the downstream Akt Ser/Thr kinase, thereby facilitating the transfer of GLUT4 from the cytoplasm to the cell membrane, enhancing cellular glucose absorption, and consequently decreasing blood glucose levels [50, 51]. Importantly, the GSK-3β could also be regulated by the PI3K/Akt pathway in the liver. Ouyang et al. reported *Ercao-Qinggan* decoction regulated apoptosis of hepatocytes in mice with acute liver failure via the PI3K/Akt/GSK3β signal pathway [52]. Similarly, Zhang et al. demonstrated that HAS ameliorates the disease progression in type 2 diabetic mice via the PI3K/Akt/GSK-3β/GS pathway, thereby reducing blood glucose and increasing glycogen synthesis [53]. Our study showed that MELNs activated the PI3K/Akt signaling and inactivated the GSK-3β. However, further research is required to elucidate the specific material basis of MELNs regulating these pathways.

## Conclusion

**Fig. 10:**
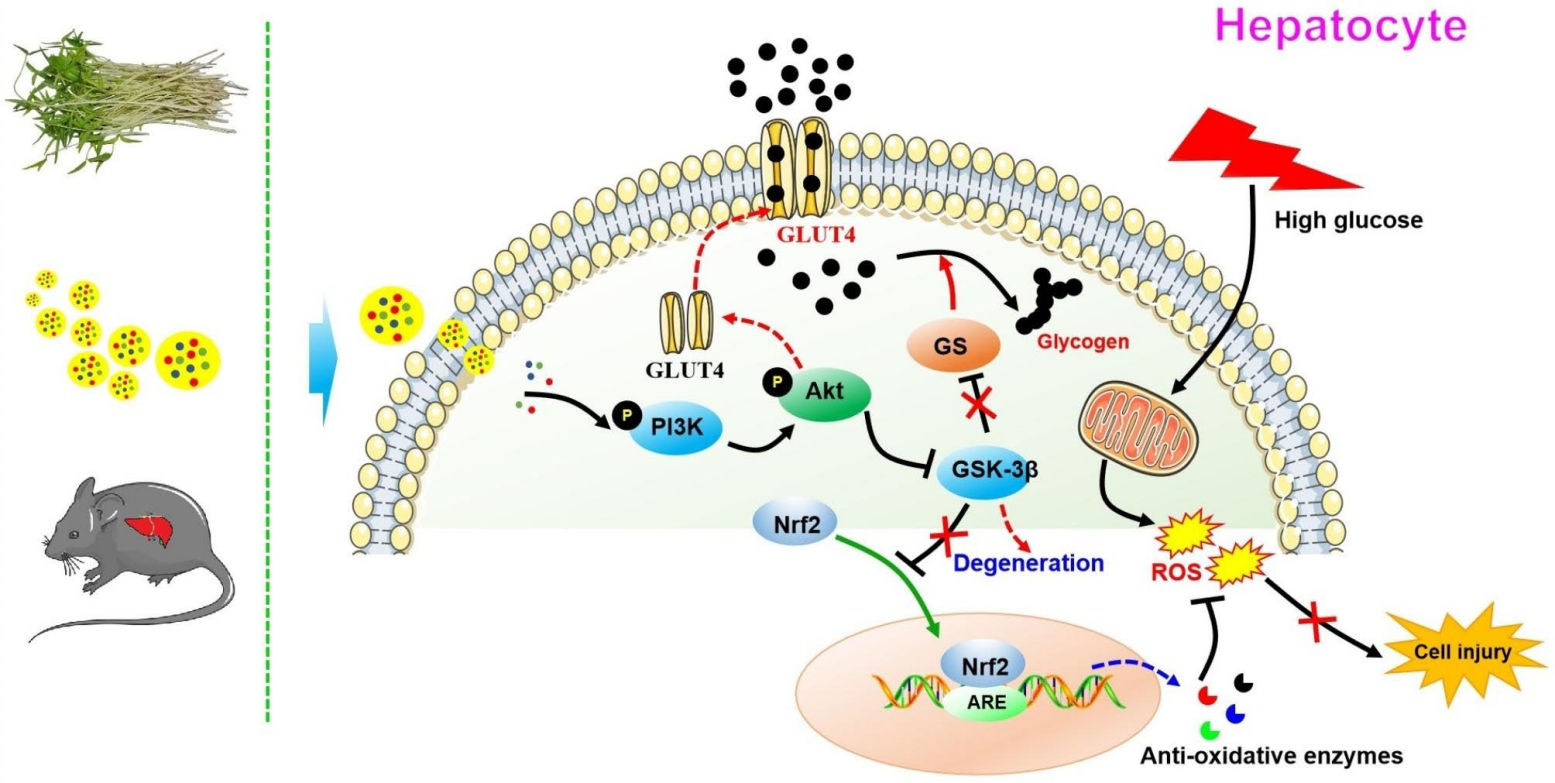
The possible molecular signaling pathway for the exosomes-like nanoparticles in mung bean sprouts against diabetic effects

Our research successfully isolated natural exosome-like nanoparticles from Mung bean sprouts (MELNs). Furthermore, our present results suggested that MELNs improved diabetic conditions in STZ/HFD mice, and the underlying molecular mechanism is related to the regulation of PI3K/Akt/GLUT4/GSK-3β signaling (Fig. 10).

## Data Availability

All data are available in the main text and are available from the corresponding authors upon reasonable request.
